# The Comparative Study of the Effectiveness of Cimetidine, Ranitidine, Famotidine, and Omeprazole in Treatment of Children with Dyspepsia

**DOI:** 10.5402/2011/219287

**Published:** 2011-04-05

**Authors:** Seyed Mohsen Dehghani, Mohammad Hadi Imanieh, Roya Oboodi, Mahmood Haghighat

**Affiliations:** Gastroenterohepatology Research Center, Pediatric Gastroenterology Department, Nemazee Hospital, Shiraz University of Medical Sciences, Shiraz 71937-11351, Iran

## Abstract

*Background*. Functional dyspepsia is a common chronic disorder with non specific upper abdominal pain or discomfort. Different approaches with anti-secretory, spasmolytic, prokinetic and anti-inflammatory effects and most preferably reduction of visceral hypersensitivity seem logical. In this study, we compared the effectiveness of the four most drugs used for treatment of dyspepsia in children. *Methods*. 169 patients between 2 to 16 years old that 47.3% was male and 52.7% was female were enrolled in this clinical trial study by the diagnosis of functional dyspepsia. Then for each patient one of the drugs; Omeprazole, Famotidine, Ranitidine or Cimetidine was administered, for a period of 4 weeks. Patients were followed after 2 and 6 weeks from the beginning of the treatment. *Results*. The distribution of drugs between these patients were including; 21.9% with Cimetidine, 21.3% with Famotidine, 30.8% with Omeperazole and 26% with Ranitidine that the proportion of patients with all symptoms relief were: 21.6% for Cimetidine, 44.4% for Famotidine, 53.8% for Omeprazole and 43.2% for Cimetidine (*P* = .024). In followups within 2 and 6 weeks after beginning medical therapy, no side effects due to drugs were seen. *Conclusion*. If a cure is defined as all symptoms relief after a period of 4 weeks treatment, our findings showed that Omeperazole are superior to Ranitidine, Famotidine, and Cimetidine for management of functional dyspepsia.

## 1. Introduction

Functional dyspepsia (FD) is a very common cause of upper gastrointestinal symptoms and discomfort [1]. FD has been defined as a functional gastrointestinal disorder (FGID) characterized by persistent or recurrent pain or discomfort centered in the upper abdomen that is not relieved by defecation or associated with changes in stool characteristics occurring at least once a week for at least 2 months in the absence of organic diseases [2]. A diagnosis of FD can be made in children mature enough to provide an accurate history of pain that is present for at least a 12-week duration, which need not be consecutive, in the preceding 12 months, the recurrent discomfort is typically centered in the upper abdomen (above the umbilicus), and there is no evidence of organic disease (including at upper endoscopy). In addition, there is no evidence that dyspepsia is exclusively relieved by defecation or is associated with the onset of a change in stool frequency or stool form. There are two presentations of functional dyspepsia, which are ulcer-like dyspepsia and dysmotility-like dyspepsia. 

The low prevalence of organic disease found in dyspepsia supports the use of reassurance and empiric therapy as initial treatment, so the Rome committee recommends that an upper gastrointestinal endoscopy should be performed in the presence of dysphagia, persistence of symptoms despite the use of acid reducing medications, or patients with recurrent symptoms after discontinuing such medications [2]. Thus, different approaches with antisecretory, spasmolytic, prokinetic, and anti-inflammatory effects, and, most preferably, reduction of visceral hypersensitivity seem logical. This could explain the variety of drugs which show a positive symptomatic response [1], currently there is no FDA-approved drug for treatment of FD [3]. 

Despite the scant evidence, anti-secretory agents are frequently recommended in the treatment of patients with a predominant complaint of pain while prokinetic agents are frequently used for bloating and early satiety [4]. Patients' symptoms that are severe enough to disrupt daily activities will likely benefit from pharmacologic therapy. Such therapy should be individualized and directed toward the predominant symptom [5]. 

For patients with predominant dyspepsia (discomfort centered in the epigastrium, nausea, early satiety, postprandial fullness, recurrent emesis), a short course of empiric therapy with an H_2_-histamine receptor antagonists or proton pump inhibitors is acceptable. There are currently no pediatric data to support the long-term benefit of anti-secretory therapy in patients with FGIDs [5].

The aim of this study is to compare the effectiveness of the commonly four most used drugs for treatment of dyspepsia in children, including Omeprazole, Ranitidine, Cimetidine, and Famotidine in a period of 4 weeks of treatment.

## 2. Patients and Methods

In this clinical trial study, 169 children between 2 and 16 years old were enrolled with the diagnosis of FD which was made by a history of recurrent or persistent abdominal pain and discomfort which was typically centered in the upper abdomen for at least a 12-week duration without any evidence of organic disorder. The other symptoms were early satiety, postprandial abdominal floating or distention, nausea and vomiting. Patients over 18 years old, and whom their medical therapy could not be completed, and those with symptoms including, fever over 38 centigrade degrees, night sweating, weight loss more than 3 kg during past month, frequent vomiting, hematochezia or hematemesis, severe localized pain, and dysphagia, were excluded from this study. Then, for each patient, one of the acid suppressant medications, Omeprazole, Famotidine, Ranitidine, or Cimetidine, was administered, for a period of 4 weeks. The drugs dosage used was as follows: Cimetidine: 10 mg/kg/dose b.i.d., Ranitidine: 1-2 mg/kg/dose b.i.d. (max: 300 mg/24 hr), Famotidine: 1 mg/kg/day b.i.d. (max: 40 mg/24 hr), and Omeprazole: 1-2 mg/kg/day q.d (max: 40 mg/24 hr). Patients were followed after 2 and 6 weeks from the beginning of the treatment. 

All the results, consisting of any probable side effects during the therapy and the effectiveness of the medication being used in treatment of the patients, were collected and analyzed. Collected data and results were analyzed by SPSS 15 software. Standard deviation, mean, and distribution of the results were also analyzed. Chi-square test and Students *t*-test were also used to analyze the results. The significant *P*-value in this study was determined to be less than  .05.

## 3. Results

There were 169 children with various dyspeptic symptoms who participated in this study. 80 patients (43.7%) were males and 89 patients (52.7%) were females. The mean age of the patients was 7.4 ± 3.2 years (range, 2–16 years). 

The mean duration of the disease among the patients was 15.9 ± 14.2 months (range, 3–60 months), and the mean of their weight was 25.6 ± 11.7 kg (range, 12–60 kg). Ninety-nine patients (58.6%) had a positive family history of FD, and 52 patients (30.8%) were passive smokers. In 108 patients (63.9%), symptoms were related to food consumption; occurrence of the symptoms in 35 patients (32.4%) was before, in 55 patients (50.9%) was after, and in 18 patients (16.7%) was both before and after food consumption. In 61 patients (36.1%), there was no relation between meal consumption and symptoms. 

Symptoms distributions are mentioned in Table 1. None of the patients had all the symptoms simultaneously. 

Of these 169 children, 37 (21.9%) patients were treated with Cimetidine, 36 (21.3%) with Famotidine, 44 (26%) with Ranitidine, and 52 (30.8%) with Omeprazole. The most common symptoms relieved regardless of type of medication were nausea (86.2%), vomiting (80.8%), and heart burn (79.5%). Abdominal pain was relieved in 63.9%. The distribution and percentage of symptoms being relieved regardless of the specific medication being administered are shown in Table 2. 

When different medications were compared, abdominal pain was improved in 45.9%, 65.9%, 66.7%, and 73.1% of Cimetidine, Ranitidine, Famotidine, and Omeprazole groups, respectively, these differences were statistically significant (*P* < .05). The distribution and percentage of symptoms being relieved in relation to the specific medication being used are mentioned in Table 3. 

The most influenced symptoms followed by medical therapy in relation with specific medication were chest pain in Famotidine group (100%), vomiting in Ranitidine group (92.7%), nausea and vomiting in Cimetidine group (90%), and nausea in Omeprazole group (87.8%), respectively. The least influenced symptoms followed by medical therapy in relation with specific medication was halitosis in all groups, 25.9%, 44.8%, 51.5%, and 52.5% in Cimetidine, Famotidine, Ranitidine, and Omeprazole groups, respectively. 

In 71 out of 169 patients (42%), all of the symptoms were relieved, not considering the specific medication being taken by them. When different medications compared 8 of 37 patients who took Cimetidine cured completely (21.6%), this cure rate in other groups were 43.2% (19 out of 44) in Ranitidine, 44.4% (16 out of 36) in Famotidine, and 53.8% (28 out of 52) in Omeprazole group, differences were statistically significant (*P* = .024).

In the followups during 2 and 6 weeks after medical therapy, no side effects due to medical therapy were seen.

## 4. Discussion

FGIDs, including functional abdominal pain (FAP), are among the most common conditions in children. A school-based study in the United States showed a 38% overall weekly prevalence of abdominal pain and persistence of symptoms for more than 8 weeks in 24% of them. Children with abdominal pain were found to miss more school than their peers, and their parents frequently missed work to take care of their children [6]. Some studies in children have shown an association between chronic or recurrent abdominal pain and higher depression and anxiety scores and poor quality of life [6]. Despite its high frequency and significant impact on quality of life of children, there is only limited evidence to support most treatments that are commonly used to treat childhood FAP. Dietary recommendations may be helpful for some patients with functional recurrent abdominal pain of childhood [7]. There are different medical therapies with different medications for treatment of this disorder in children. In FD, the placebo response has varied form 13–73% [8]. Patients' symptoms that are severe enough to disrupt daily activities will likely benefit from pharmacologic therapy [8]. Such therapy should be individualized and directed toward the predominant symptom [8]. Treatment modalities include medications, diet modification, herbal preparations, and behaviorally psychologic interventions [9]. Enteric-coated peppermint-oil capsules, believed to exert calcium channel blockade in smooth muscle, were shown in a randomized, placebo-controlled study to decrease the severity of abdominal pain, but not other symptoms in pediatric patients with irritable bowel syndrome [9]. Pharmacotherapy for treatment of FGIDs consists of anticholinergic agents, tricyclic antidepressants, serotonergic agents, selective serotonin reuptake inhibitors, 5-HT_3_ receptor antagonists, 5-HT4 receptor agonists, and acid suppressive therapy [5]. For patients with predominant dyspepsia (discomfort centered in the epigastrium, nausea, early satiety, postprandial fullness, recurrent emesis), a short course of empiric therapy with H_2_-receptor antagonists or proton pump inhibitors is acceptable [5]. Some meta-analysis studies showed that H_2_-receptor antagonists did or did not have a significant therapeutic effect in FD [10, 11]. A meta-analysis of randomized controlled clinical trials has shown that there may be a benefit in the use of H_2_-receptor antagonists in patients suffering from FD [12]. In another study, it was found that Famotidine was equally effective as placebo [6].

In a meta-analysis, proton pump inhibitors were regarded as superior to H_2_-receptor antagonists and antacids in patients with “noninvestigated” dyspepsia [13], H_2_-receptor antagonists and antacids showed positive effects in approximately 40% of patients (which is in the range of the placebo response rate) whereas proton pump inhibitors response rates were significantly higher, adding an additional 20% [1]. In two preliminary studies of Omeprazole, a proton pump inhibitor, for the treatment of nonulcer dyspepsia, only 50% of the patients treated with Omeprazole had a response, as compared with 25% of those receiving placebo [14]. In a double-blind randomized placebo-controlled study of 4 weeks of Lansoprazole (a proton pump inhibitor) for the treatment of FD in Chinese patients, findings implicated that proton pump inhibitors treatment was not superior to placebo for the management of FD in Chinese patients [15]. Proton pump inhibitors especially improved the symptoms of epigastric pain and heart burn [1]. Several studies in the primary care setting have concluded that proton pump inhibitors are more effective than H_2_-receptor antagonists or antacids in treating heart burn and dyspeptic symptoms [16]. Therefore, empiric acid suppression would seem to be the favored management approach for the treatment of FD [17]. 

Since the various proton pump inhibitors are of equivalent efficacy and safety, the cost and acceptability of a particular proton pump inhibitor preparation may be more important when selecting among them than comparable efficacy [18]. 

In this study, we compared the effectiveness of four medications including Cimetidine, Famotidine, Ranitidine (all of them H_2_-receptor antagonists), and Omeprazole (a proton pump inhibitor), for treatment of children with dyspeptic symptoms, to find the best one for this reason. So if a cure is defined as all symptoms relief after a period of 4 weeks treatment, our analysis indicates that there is a significant difference between response rate and the specific medication being used (*P* = .024), and it reveals that the most effective medication, when considering cure as all symptoms being relieved, was Omeprazole with response rate of 53.8% and then with Famotidine (44.4%), Ranitidine (43.2%), and, at last, with Cimetidine (21.6%). Although no significant difference (*P* = .06) was found in abdominal pain relief in relation with specific medication consumption, but due to higher response to Omeprazole, it seems that Omeprazole was better than others, and Cimetidine had the least effect. Also, there were no significant differences between other symptoms relief (*P* > .05) and the specific medication taken by the patients, except for epigastric pain which responds significantly (*P* = .018) to Famotidine and Ranitidine with response rate of 68.4% then Omeprazole with 66.7% and at last Cimetidine with 28.6%.

According to our results and the fact that no significant side effects being detected, and also due to the fact that Ranitidine and Omeprazole were the most effective medications on only one of the symptoms (epigastric pain) comparing with Omeprazole that had the best result on all symptoms being relieved, it cannot be concluded that Ranitidine and Famotidine have equal or better effect in treatment of FD, but, in fact, the best medical therapy for treatment of FD is Omeprazole, or in another way Omeprazole is superior to H_2_-receptor antagonists for treatment of FD.

At the end it is important to note that since 3 of 4 medications that we used in our study had approximately an equivalent efficacy and safety, the cost of a particular medication may be more important, when selecting among them, than comparable efficacy.

## Figures and Tables

**Table 1:** Distribution of symptoms in all patients.

Symptom	Distribution	Percentage
Abdominal pain	169	100
Halitosis	129	76.3
Nausea	87	51.4
Anorexia	87	51.4
Nocturnal awakening	76	44.9
Early satiety	54	31.9
Vomiting	47	27.8
Heart burn	39	23.1
Chest pain	19	11.2

**Table 2:** Distribution and percentage of symptoms being relieved regardless of the specific medication.

Symptom	Distribution before treatment	Symptoms being relieved	Percentage of symptoms being relieved
Nausea	87	75	86.2
Vomiting	47	38	80.8
Heart burn	39	31	79.5
Nocturnal awakening	76	54	71
Chest pain	19	13	68.4
Early satiety	54	35	64.8
Abdominal pain	169	108	63.9
Anorexia	87	46	52.9
Halitosis	129	58	44.9

**Table 3:** Distribution of symptoms being relieved in relation to specific medication.

Symptoms	Patients	Drug	Patients before treatment	Percentage before treatment	Patients whom their symptoms relieved	Percentage of whom their symptoms relieved	*P*-value*
Abdominal pain	169	Cimetidine	37	21.9	17	45.9	<.05
		Famotidine	36	21.3	24	66.7	
		Omeprazole	52	30.8	38	73.1	
		Ranitidine	44	26	29	65.9	

Epigastric pain	89	Cimetidine	21	23.6	6	28.6	<.05
		Famotidine	19	21.3	13	68.4	
		Omeprazole	30	33.7	20	66.7	
		Ranitidine	19	21.3	13	68.4	

Periumbilical pain	95	Cimetidine	20	21	11	55	>.05
		Famotidine	17	17.9	11	64	
		Omeprazole	31	32.6	23	74	
		Ranitidine	27	28.5	16	59	

Nausea	87	Cimetidine	20	23	18	90	>.05
		Famotidine	13	15	11	84	
		Omeprazole	33	38	29	87.8	
		Ranitidine	21	24	17	80.9	

Vomiting	47	Cimetidine	10	21.3	9	90	>.05
		Famotidine	8	17	6	75	
		Omeprazole	17	36.2	12	70.6	
		Ranitidine	12	25.5	11	91.7	

Anorexia	87	Cimetidine	18	20.7	8	44.4	>.05
		Famotidine	24	27.6	12	50	
		Omeprazole	24	27.6	13	54.2	
		Ranitidine	21	24	13	61.9	

Early satiety	54	Cimetidine	15	27.8	10	66.7	>.05
		Famotidine	12	22	6	50	
		Omeprazole	13	24	11	84.6	
		Ranitidine	14	25.6	8	57.1	

Heart burn	39	Cimetidine	6	15.4	3	50	>.05
		Famotidine	8	20.5	7	87.5	
		Omeprazole	19	48.7	16	54.2	
		Ranitidine	6	15.4	5	83.3	

Chest pain	19	Cimetidine	5	26.3	2	40	>.05
		Famotidine	2	10.5	2	100	
		Omeprazole	8	42	6	75	
		Ranitidine	4	21	3	75	

Halitosis	129	Cimetidine	27	21	7	25.9	>.05
		Famotidine	29	22.5	13	44.8	
		Omeprazole	40	31	21	52.5	
		Ranitidine	33	25.6	17	51.5	

Nocturnal awakening	76	Cimetidine	16	21	9	56.3	>.05
		Famotidine	18	23.7	14	77.7	
		Omeprazole	24	31.6	18	75	
		Ranitidine	18	23.7	13	72.2	

*The *P*-value is indicative of comparison between various medications effectiveness with each symptom relief.
