# Erratum to “The Comparative Study of the Effectiveness of Cimetidine, Ranitidine, Famotidine, and Omeprazole in Treatment of Children with Dyspepsia”

**DOI:** 10.1155/2013/206546

**Published:** 2013-03-28

**Authors:** Seyed Mohsen Dehghani, Mohammad Hadi Imanieh, Roya Oboodi, Mahmood Haghighat

**Affiliations:** Gastroenterohepatology Research Center, Pediatric Gastroenterology Department, Nemazee Hospital, Shiraz University of Medical Sciences, Shiraz 71937-11351, Iran

In the paper “*The comparative study of the effectiveness of Cimetidine, Ranitidine, Famotidine, and Omeprazole in treatment of children with dyspepsia*” by S. M. Dehghani et al., published in ISRN Pediatrics, Volume 2011, Article ID 219287, one sentence needs to be amended as it is incorrect or incomplete. In the abstract, in line 9, the correct sentence should read “21.6% for Cimetidine, 43.2% for Ranitidine, 44.4% for Famotidine, and 53.8% for Omeprazole (*P* = .024).”

